# Correction to ‘Stabilization of SMAR1 mRNA by PGA2 involves a stem–loop structure in the 5′ UTR’

**DOI:** 10.1093/nar/gkac168

**Published:** 2022-03-14

*Nucleic Acids Research*, Volume 35, Issue 18, 15 September 2007, Pages 6004–6016, https://doi.org/10.1093/nar/gkm649

The authors regret the presence of undisclosed image splicing in Figure 5, panels D and E of their article. The authors also wish to report four image assembly errors in Figure 5, panels D and E as detailed below. The errors have most likely arisen while working with multiple files with different exposures.

While the authors do not have access to the original raw data which was generated over 17 years ago, the same experimental data in its earliest form, was included in the thesis of one of the authors, Dr. Shravanti Rampalli ‘Regulation of Retroviral and Eukaryotic Transcription through MAR sequences and MAR binding protein SMAR1’ submitted to the University of Pune, India in June 2005. This information is used here to update Figures 5D and 5E.

**Error 1:**

- Figure 5D, lanes 5–7 and Figure 5E, lanes 13–15 are duplicate images. Figure 5D, lanes 5–7 is incorrect. The correct image is shown in the thesis in panel B, PGA2, SMAR1.

- Figure 5D, lanes 11–13 and Figure 5E, lanes 1–3 are duplicate images. Figure 5D, lanes 11–13 is incorrect. The correct image is shown in the thesis in panel B, PGA2, HDAC1.

**Error 2:** Figure 5D, lanes 6–7 and lanes 12–13, the labels for the test siRNA and scrambled siRNA were swapped during image assembly.

**Error 3:** Figure 5D, lanes 15–16, the labels were swapped during figure assembly.

**Error 4:** Figure 5E, lanes 16–18 and Figure 5D, lanes 14–17 are duplicate images. Figure 5E, lanes 16–18 is incorrect. Experiments involving data for Figure 5E lanes 16–18 were completed after the thesis was submitted. The uncropped gel image is provided below. The correct gel image corresponding to Probe III (Figure 5E lanes 16–18) has been included in the corrected Figure 5E below. Since this is a different experiment, the signal intensity varies from the previously published subpanel.


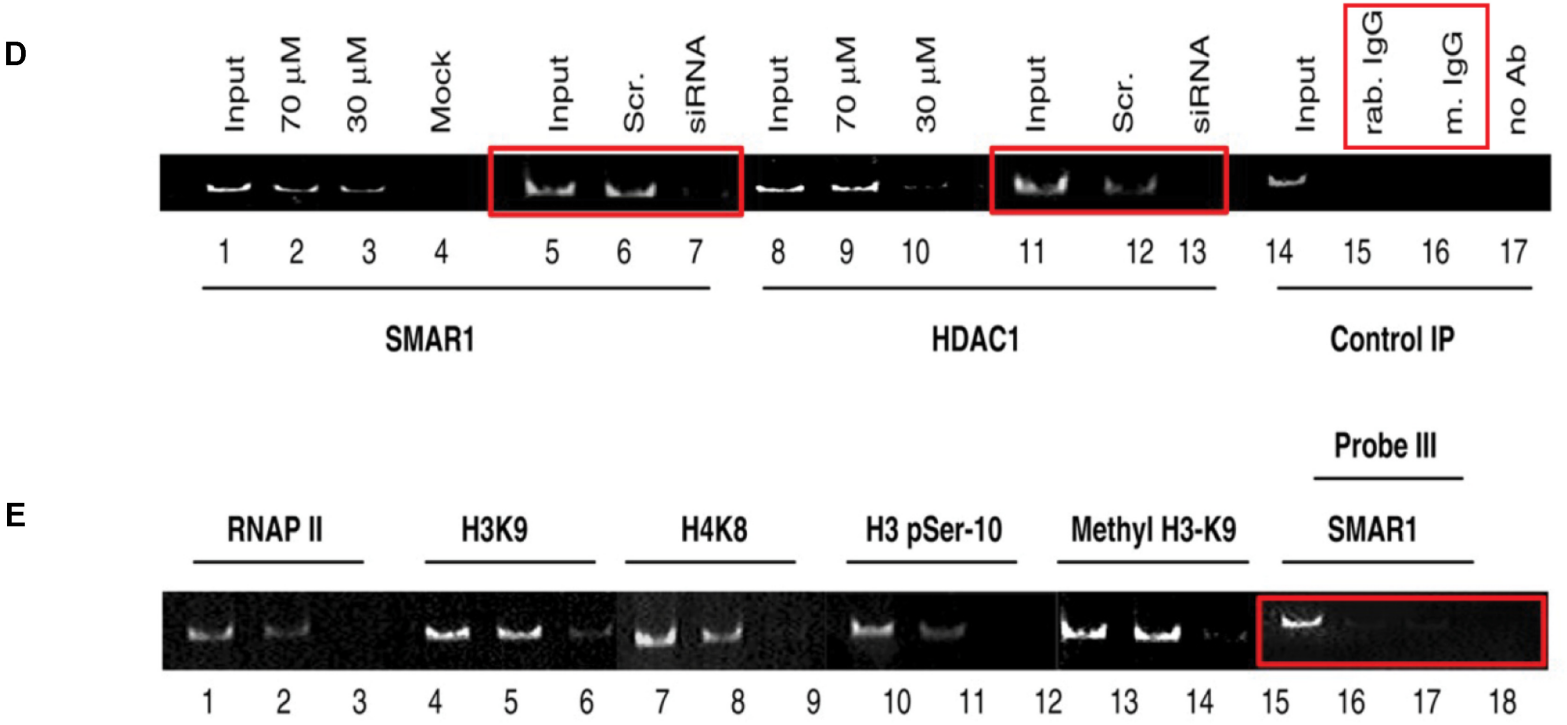


**Image depicting the assembly errors** marked in red boxes, in Figures 5D and 5E.


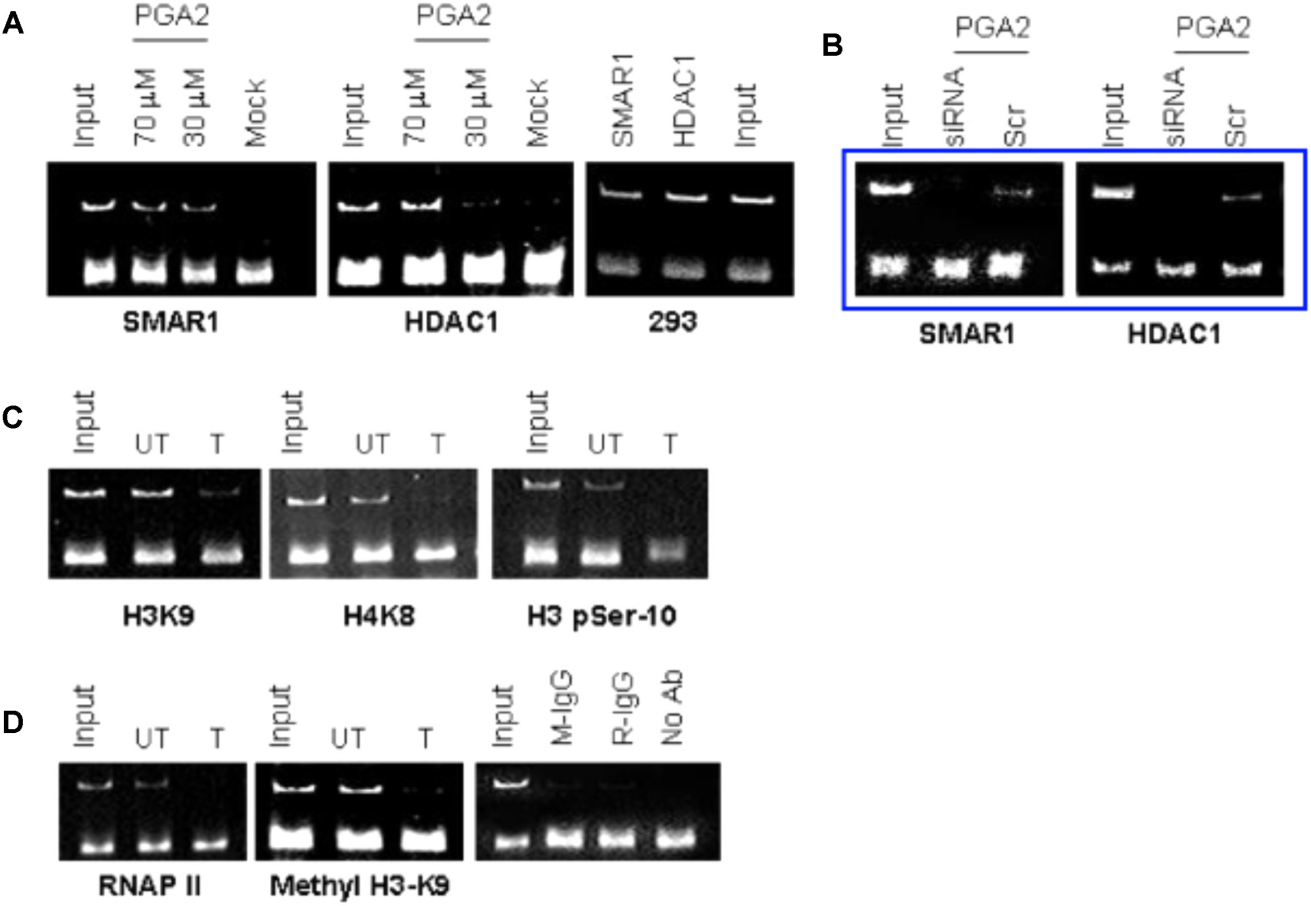


**Original data as presented in the thesis** (showing the panels used for assembly): ChIP samples highlighted in blue box are from the thesis and correspond to Figure 5D lanes 5–7 and 11- 13, which should have been included in the article figure. The thesis panel A and panel D (subpanel 3) are correctly included in the article Figure 5D as lanes 1–4 (SMAR1 ChIP after PGA2 treatment) and lanes 8–10, respectively (HDAC1 ChIP after PGA2 treatment). In all panels, the lower strong bands which correspond to primer dimers were not included during image assembly. This was done as a standard procedure to improve comprehension. The article does not include subpanel (293) from Panel A.


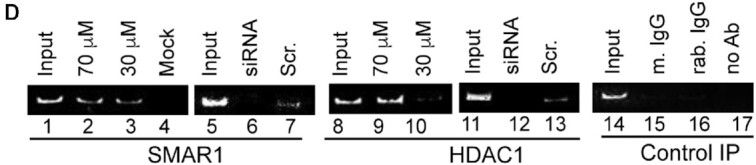


**Corrected Figure 5D**. The correct panels for the siRNA experiments have now been provided in the corrected Figure 5D. Since these are separate experiments the signal intensities differ.


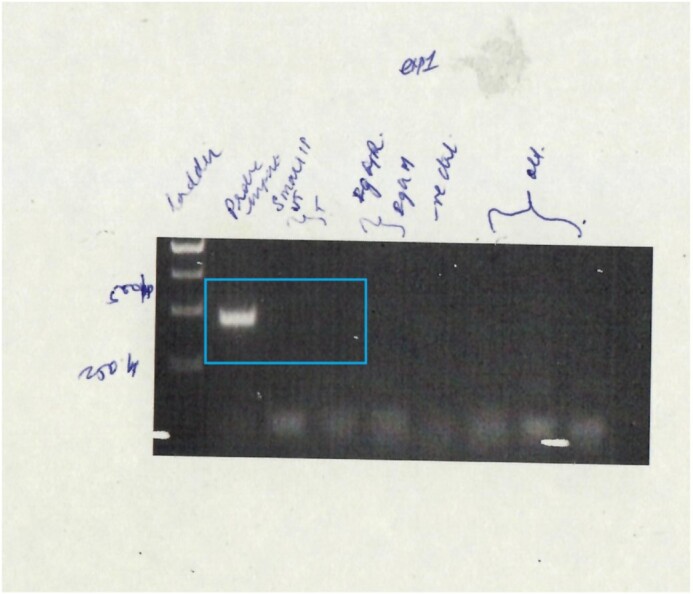


**Figure 5E, lanes 16–18, original data:** The ChIP data indicated in the blue box corresponds to Figure 5E, lanes 16–18, which should have been included in the article figure. Panel C, and panel D, subpanels 1 and 2 were used correctly to assemble the rest of Figure 5E.


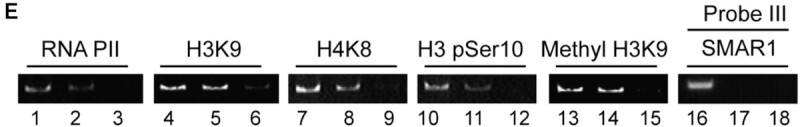


**Corrected Figure 5E**.


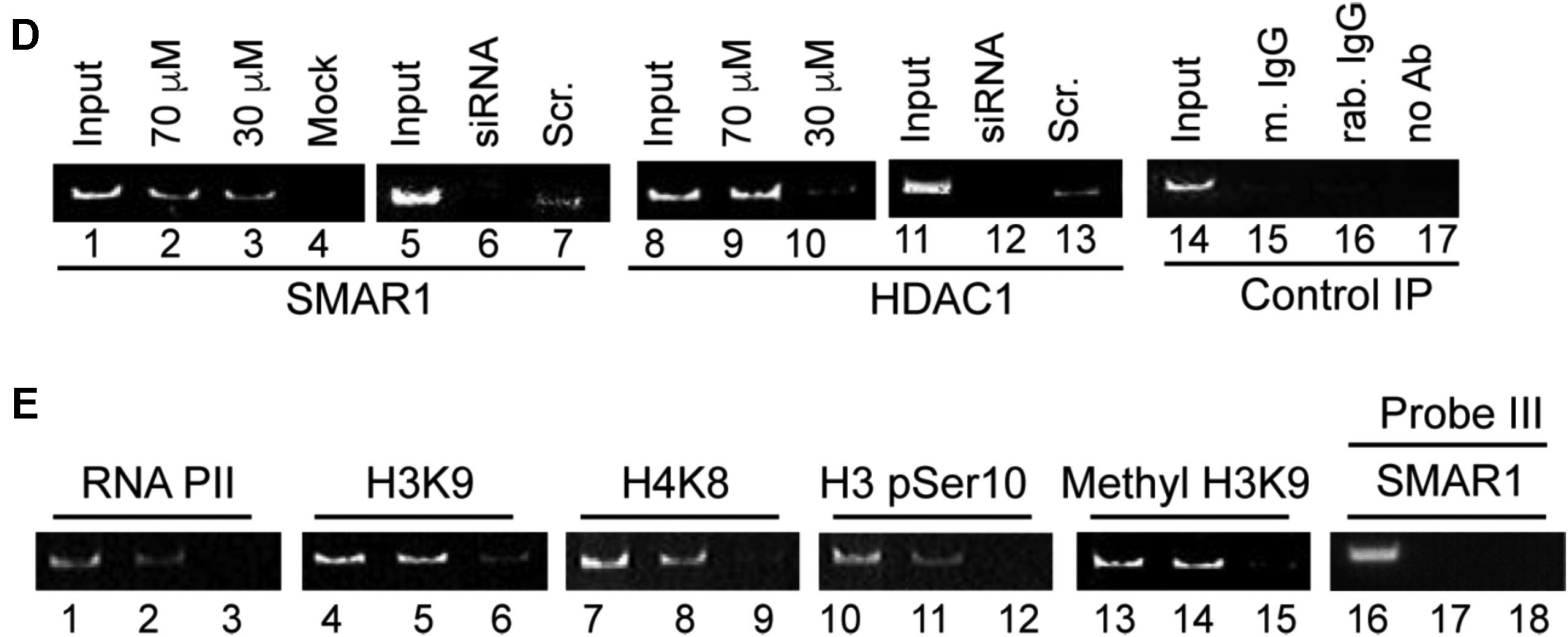


**Corrected Figure 5D-E:** This new figure 5D contains the replaced panels for SMAR1 and HDAC1 ChIP upon SMAR1 and scrambled siRNA after PGA2 treatment. The new figure 5E contains the replaced ChIP image for SMAR1 occupancy on probe III for both treatment conditions.

The interpretation of the data and the conclusions of the article are unaffected. However, the authors sincerely apologise to the readers and publisher for the inaccuracies.

